# *Helicobacter pylori* and Its Antibiotic Heteroresistance: A Neglected Issue in Published Guidelines

**DOI:** 10.3389/fmicb.2019.01796

**Published:** 2019-08-13

**Authors:** Albert A. Rizvanov, Thomas Haertlé, Lydia Bogomolnaya, Amin Talebi Bezmin Abadi

**Affiliations:** ^1^Institute of Fundamental Medicine and Biology, Kazan Federal University, Kazan, Russia; ^2^Biopolymers Interactions Assemblies, Institut National de la Recherche Agronomique, Nantes, France; ^3^Department of Animal Nutrition and Feed Management, Poznan University of Life Sciences, Poznań, Poland; ^4^Institute of Biochemistry and Biophysics, University of Tehran, Tehran, Iran; ^5^Department of Microbial Pathogenesis and Immunology, Texas A&M University Health Science Center, Bryan, TX, United States; ^6^Department of Bacteriology, Faculty of Medical Sciences, Tarbiat Modares University, Tehran, Iran

**Keywords:** *Helicobacter pylori*, heteroresistance, antibiotic resistance, guidelines, antibiotic therapy

## Abstract

“Heteroresistance” is a widely applied term that characterizes most of the multidrug-resistant microorganisms. In microbiological practice, the word “heteroresistance” indicates diverse responses to specific antibiotics by bacterial subpopulations in the same patient. These resistant subpopulations of heteroresistant strains do not respond to antibiotic therapy *in vitro* or *in vivo*. Presently, there is no standard protocol available for the treatment of infections caused by heteroresistant *Helicobacter pylori* in clinical settings, at least according to recent guidelines. Thus, there is a definite need to open a new discussion on how to recognize, how to screen, and how to eliminate those problematic strains in clinical and environmental samples. Since there is great interest in developing new strategies to improve the eradication rate of anti-*H. pylori* treatments, the presence of heteroresistant strains/clones among clinical isolates of the bacteria should be taken into account. Indeed, increased knowledge of gastroenterologists about the existence of heteroresistance phenomena is highly required. Moreover, the accurate breakpoints should be examined/determined in order to have a solid statement of heteroresistance among the *H. pylori* isolates. The primary definition of heteroresistance was about coexistence of both resistant and susceptible isolates at the similar gastric microniche at once, while we think that it can be happened subsequently as well. The new guidelines should include a personalized aspect in the standard protocol to select a precise, effective antibiotic therapy for infected patients and also address the problems of regional antibiotic susceptibility profiles.

## Introduction

*Helicobacter pylori* (*H. pylori*) is a Gram-negative and transmissible microorganism that resides in the deep gastric mucosa of humans. Current estimation of people infected with this microorganism is around 4.4 billion of the world’s population (Kusters et al., 2006). The infection causes serious digestive disease such as chronic gastritis, peptic ulcer disease (PUD), and gastric cancer in 1–10% of colonized individuals (Blaser, 1990, 1992; Parsonnet et al., 1991; Atherton, 2006). It is also suspected of inducing or worsening Parkinson’s disease (Dobbs et al., 2016). For reasons that are unclear, many of the colonized persons remain asymptomatic for their whole life (Malfertheiner et al., 2018b). Treating *H. pylori* infection induces the regression of mucosa-associated lymphoid tissue (MALT) lymphoma and has recently been considered as a preventive approach for the management of gastric cancer (Bayerdörffer et al., 1995; Wong et al., 2004; Atherton, 2006). The elimination of this pathogen is also reducing or eliminating Parkinson’s disease symptoms (Liu et al., 2017). Many guidelines have been established that outline a number of therapeutic regimens to provide optimal treatments using conventional antibiotics and proton pump inhibitors (PPIs). However, the lack of an ideal therapy is still a major challenge in *H. pylori*-related treatment. Apart from the many antibiotics applied to fight the infection, a skyrocketing increase in antimicrobial resistance is hampering the elimination of *H. pylori* (Chiba et al., 1992; Gao et al., 2010). Although patient compliance and other environmental factors are partially involved, antibiotic resistance is mainly responsible for *H. pylori* treatment failures (Graham, 1998; Björkholm et al., 2001; De Francesco et al., 2010; Graham and Fischbach, 2010; Thung et al., 2016). Prevalence of antibiotic resistance among *H. pylori* strains varies in different geographic areas (Björkholm et al., 2001; Glupczynski et al., 2001; Megraud, 2004; Fischbach and Evans, 2007; Gao et al., 2010; Megraud et al., 2012; Khaiboullina et al., 2016). This global problem has triggered a universal drive to find a better solution. Following the rapid increase in resistance rates for conventional antibiotics used for eliminating *H. pylori*, many studies showed failures of first, second, and third lines of treatments (Wolle et al., 2002; Zullo et al., 2007; Kim et al., 2011; Oleastro et al., 2011; Saracino et al., 2012; Tonkic et al., 2012; Hsiang et al., 2013; Maleknejad et al., 2015; Kori et al., 2017; Macias-Garcia et al., 2017). The ultimate goal in therapeutic regimens against *H. pylori* is to achieve more than 90% eradication rate, which has currently proved unreachable (Zagari et al., 2018). Most recent worldwide reports together with meta-analyses indicate that the efficacy of antibiotics available to treat *H. pylori* infections has been significantly reduced (Figueiredo et al., 2005; Suzuki and Mori, 2018). In addition, emergence of multidrug-resistant *H. pylori* strains has been devastating for clinicians and microbiologists aiming to eliminate this infection (Kwon et al., 2003; Boyanova, 2009). The situation is made worse by the lack of a vaccine against *H. pylori* (Luo et al., 2018; Pan et al., 2018; Malfertheiner et al., 2018a,b). Now that antibiotic resistance has been recognized as a growing problem in the treatment of *H. pylori* infection, a much less investigated phenomenon among bacteria, namely, “heteroresistance” should be addressed. An understanding of the causes of increasing prevalence of resistant strains or, in other words, heteroresistant strains, could be pivotal in the design of effective guidelines both on national and international scales. In this paper, we discuss first the general concept of heteroresistance reported for some *H. pylori* strains; second, we will compare this resistance to co-infection with this bacterium. Ultimately, we hope for a reformulation of the treatment options of antibiotic-resistant *H. pylori* infections.

The phenomenon of heteroresistance is based on the growth differences in bacterial subpopulations within the same strain in response to a particular antibiotic (El-Halfawy and Valvano, 2013; Wang et al., 2014; El-Halfawy and Valvano, 2015). “Heteroresistance” can be one of several terms applied to multidrug-resistant microorganisms (Alam et al., 2001; Wannet, 2002; Plipat et al., 2005; Matteo et al., 2006; Hawley et al., 2008; Goldman et al., 2014; He et al., 2017). Heteroresistant clones are able to survive in the presence of antibiotics in both *in vitro* and *in vivo* microniches (Yeldandi et al., 1988; Gillespie, 2002; Ribes et al., 2010; Russo et al., 2011). Due to the lack of standard methods of characterizing heteroresistance, its detection is poor (Hofmann-Thiel et al., 2009; Huang et al., 2010; Goldman et al., 2014; He et al., 2017). The first heteroresistant Gram-negative bacterium, *Haemophilus influenzae,* was discovered in 1947 (Alexander and Leidy, 1947). Since then, not many bacteria have been listed as heteroresistant, even though this phenomenon is widely spread across many bacterial species. The Clinical and Laboratory Standards Institute (CLSI), one of several international bodies dealing with antimicrobial resistances, has published many reports determining resistant, sensitive, and intermediately resistant organisms. However, there is no established definition of heteroresistant strains (Osato et al., 2001; El-Halfawy and Valvano, 2015). Additionally, practitioners are not fully aware of the frequency and clinical activity of heteroresistant isolates. Therefore, the focus of this paper is to pinpoint the clinical impact of *H. pylori* heteroresistant strains and to highlight the urgent need for revised guidelines to manage and cure this infection.

## Heteroresistant *H. Pylori*

The emergence of heteroresistance in *H. pylori* resistant strains has never been discussed in published guidelines (Malfertheiner et al., 2007, 2012; Graham and Shiotani, 2008; Fock et al., 2013; Subspecialty Group of Gastroenterology, 2015; Sugano et al., 2015; Zagari et al., 2015; Chey et al., 2017; Mahachai et al., 2017; Smith et al., 2017). Matteo et al. found two *H. pylori* strains that significantly differed in antibiotic sensitivity even though they were obtained from two antral biopsies isolated from a single patient (Matteo et al., 2008). The minimum inhibitory concentration (MIC) for amoxicillin in those strains varied between 2 and 0.06 μg/ml, respectively. This finding brings a new insight about the importance of heteroresistant strains and the need for their detection prior to antibiotic prescription. The important clinical consequence of the existence of heteroresistant *H. pylori* strains is the possibility of their further propagation despite antibiotic therapy. The absence of an accurate and rigorous approach hampers the exact determination of the real prevalence of these strains in the affected individuals. Additionally, data about persistence or virulence of these heteroresistant bacteria are currently lacking (El-Halfawy and Valvano, 2013; Didelot et al., 2016; Halaby et al., 2016). In consideration of the likely failure of treatment of heteroresistant strains, the rapid increase of gastroduodenal diseases is both predictable and expected. Consequently, the high potential of heterogeneity reported for this bacterium must be thoroughly researched (Yamaoka, 2012).

## Heteroresistant Strains Versus Co-Infections

There is a common belief among microbiologists about the existence of two or more different *H. pylori* strains colonizing the same human stomach (Jorgensen et al., 1996; Kersulyte et al., 1999; Lai et al., 2016; Mansour et al., 2016; Raymond et al., 2016). It seems that following bacterial colonization of our stomach, *H. pylori* is able to use its remarkable genetic variability to create new variants (Jiang et al., 1996; Gottke et al., 2000; Spechler et al., 2000; Suerbaum, 2000; Gravina et al., 2016). Given the large potential of *H. pylori* in generating new genetically diverse isolates, the co-infections theory, in our opinion, needs to be updated by the incorporation of the heteroresistance concept. According to the theory of heteroresistance, human gastric mucosa can be a territory for both resistant and sensitive *H. pylori* strains exposed to specific antibiotics while the origin of isolates remains identical. During infections, approximately 5% of them are actually mixed infections caused by independent *H. pylori* strains. As such, evolved pathogen population is composed of both genetically identical and different strains (Lai et al., 2016). It is important to investigate if heteroresistant strains affect the final outcome (severe or mild diseases) of this infection or not. Additionally, the exact prevalence of co-infections and heteroresistance *in vivo* should be investigated in greater detail in future studies. Clearly, both phenotypical and genotypical analyses are required to answer this difficult question.

## An Explanation for Inconsistent Findings in *in vitro* and *in vivo* Experiments

Currently, we are still far from understanding the exact mechanisms involved in the development of this less-recognized biologic action among the bacteria. Clinicians are well aware of inconsistencies between *in vitro* and *in vivo* susceptibility tests (Glupczynski, 1993; Best et al., 1997; Loo et al., 1997; Bereswill et al., 1999; Chatsuwan and Amyes, 1999; van der Voort et al., 2000; Gonzalez et al., 2001; Warburton-Timms and McNulty, 2001; Adeniyi et al., 2009). Unfortunately, there is no universally standard method to determine MIC for *H. pylori* species. We suggest that heteroresistance is a likely cause of this inconsistency. Our main evidence for this claim is the concept of the simultaneous presence of various *H. pylori* genotypes in the human stomach that are associated with different susceptibility profiles (Kim et al., 2003; Graham and Fischbach, 2010; Lee et al., 2014). Because independent subpopulations of *H. pylori* are growing rapidly, we detect numerous different isolates with confusing susceptibility patterns originating from the same patient. Propagation of various *H. pylori* isolates *in vitro* can lead to conflicting results of antibiotic susceptibility testing. Thus heteroresistance is a major clinical issue, which should attract very intense attention in the upcoming years.

## New Guideline Against *H. Pylori*?

Similar to other bacterial infections, *H. pylori* management was a challenging area of the research for decades after its discovery (Malfertheiner et al., 2007, 2012; Subspecialty Group of Gastroenterology, 2015; Zagari et al., 2015; Isaeva et al., 2016; Authors, Responsible in representation of the DGVS, 2017; Chey et al., 2017; Mahachai et al., 2017; Smith et al., 2017). As far as diagnosis is concerned, there is reasonable consensus between scientists. However, there is absolutely no agreement on the optimal therapeutic intervention especially in the case of first-line treatment (Chey et al., 2007; Malfertheiner et al., 2007, 2012, 2017; Asaka et al., 2010). Overall, the mechanisms of resistance to all antibiotics used against *H. pylori* are well known (Debets-Ossenkopp et al., 1996; Burns et al., 1998; Megraud, 1998, 2003; van der Wouden et al., 2001; Keating, 2013). The current body of evidence is based on the fact that many antibiotic therapy failures are the result of genetic variations among the different *H. pylori* isolates that cause mixed infections in a host. The open question, however, is about the impact of heteroresistance on the development of severe gastroduodenal disorders in infected patients. Indeed, a long list of experiments is necessary to pinpoint the actual role of heteroresistance in the development of *H. pylori* persistent infections. Thus, updated guidelines would lead to a more effective cure if they would consider the practical approaches in dealing with heteroresistant strains. As such, this paper can be useful in the preparation for next Maastricht meetings aimed to enrich the content of current guidelines with the concept of heteroresistant *H. pylori* strains.

## Future Perspectives of *H. Pylori* Treatment

Antibiotic resistance among clinical *H. pylori* isolates is rapidly disseminating worldwide and we have to think deeply how to better manage it. At present, low doses of prescribed antibiotics, slow bacterial growth rate, bacterial coccoid forms, and genetic mutations in *H. pylori* are the major reasons for antibiotic resistance reported so far. Research into the presence of heteroresistance isolates of this bacterium been sorely neglected. Integration of heteroresistance phenomenon in *H. pylori* in clinical treatment decisions is essential. The primary definition of heteroresistance was about coexistence of both resistant and susceptible isolates at the similar gastric microniche at once, while we think that it can be happened subsequently as well. Currently, there are no data describing in detail the heteroresistant *H. pylori* strains, which reduces the usefulness of the current guidelines. We firmly believe that this is the time to improve our understanding of the heteroresistance phenomena and incorporate this into uniform guidelines. Furthermore, there is a definite need to initiate discussions and learn how to recognize, manage, screen, and eliminate these infectious strains from clinics and the environment. Undoubtedly, there is an urgent necessity for new guidelines describing the heteroresistance phenomena of *H. pylori* strains.

## Conclusion

To the best of our knowledge, this is the first paper suggest new challenge for designing the new useful guideline according to the evidence-based findings about the antibiotic resistance. Indeed, increased knowledge of gastroenterologists about the existence of heteroresistance phenomena is highly required. Moreover, the accurate breakpoints should be examined/determined in order to have a solid statement of heteroresistance among the *H. pylori* isolates.

## Author Contributions

All authors listed have made a substantial, direct and intellectual contribution to the work, and approved it for publication.

### Conflict of Interest Statement

The authors declare that the research was conducted in the absence of any commercial or financial relationships that could be construed as a potential conflict of interest.
